# A Review on Electrochemical Sensors and Biosensors Used in Chlorogenic Acid Electroanalysis

**DOI:** 10.3390/ijms222313138

**Published:** 2021-12-05

**Authors:** Irina Georgiana Munteanu, Constantin Apetrei

**Affiliations:** Department of Chemistry, Physics and Environment, Faculty of Sciences and Environment, “Dunărea de Jos” University of Galaţi, 47 Domneasca Street, 800008 Galaţi, Romania; georgiana.munteanu@ugal.ro

**Keywords:** chlorogenic acid, electrochemical sensor, enzyme, biosensor, antioxidant activity

## Abstract

Chlorogenic acid (5-O-caffeoylquinic acid) is a phenolic compound from the hydroxycinnamic acid family. Epidemiological, biological, and biochemical studies concur to support the beneficial role of chlorogenic acid in human health, along with other dietary phenolic compounds. Thus, chlorogenic acid has been reported to exert inhibitory effects on carcinogenesis in the large intestine, liver, and tongue, and a protective action on oxidative stress in vivo, together with anti-inflammatory, antidiabetic and antihypertensive activities. It is also claimed to have antifungal, antibacterial and antiviral effects with relatively low toxicity and side effects, alongside properties that do not lead to antimicrobial resistance. Due to its importance, numerous methods for determining chlorogenic acid (CGA), as well as for its derivatives from coffee beans and other plants, were elaborated. The most frequently used methods are infrared spectroscopy, high performance liquid chromatography (HPLC), capillary electrophoresis, liquid chromatography-mass spectrometry and chemiluminescence. Although these methods proved to be efficient for quantifying CGA and its derived products, a number of deficiencies were identified: they are time consuming, laborious, and require expensive instruments. Therefore, electrochemical methods have been developed and used in the determination of CGA in different nutraceuticals or food products. The present review aims to present the main progresses and performance characteristics of electrochemical sensors and biosensors used to detect CGA, as it is reported in a high number of relevant scientific papers published mainly in the last decade.

## 1. Introduction

Currently, the popularity of healthy foods has led to better studies on the impact of phytochemical compounds. These represent one of the richest reservoirs of organic compounds with various structures, called secondary metabolites, unique for each individual species [1].

Unfavorable conditions for plants, such as extreme temperature, drought, heavy metals, nutritious deficiencies and high salinity, generate high concentrations of reactive oxygen species (ROS), which can produce oxidative stress. To avoid this, cells have a complex oxidative system, which contains enzymatic and non-enzymatic elements. The molecules of the non-enzymatic system have different action mechanisms, such as inhibition of enzyme activity, chelation of the trace elements involved in producing free radicals, absorption and inactivation of reactive species, or increase of protection through other means of antioxidant defense [2].

From among the thousands of secondary compounds present in plants, phenolic compounds play a fundamental role against oxidative stress, representing one of the most frequently synthesized and studied classes [3]. They may be found in various parts of plants, such as roots, leaves, flower petals, as well as in fruit skins, seeds and pulp, which are consumed by people or used as part of nutraceutical formulas [4]. Phenolic compounds are considered genuine antioxidants, being associated with benefits in terms of decreasing the risks related to the onset of chronic diseases, as has been demonstrated by numerous reviews on their biological activity [5,6].

Phenolic compounds are metabolites frequently present in various plant sources, and play a major role in human health due to their antioxidant activity and their anti-inflammatory, antimicrobial and anticarcinogenic properties [7]. As antioxidants, these compounds can protect the cellular constituents against oxidative deterioration and, therefore, limit the risk of the onset of various degenerative diseases associated with oxidative stress [8]. They are capable of neutralizing free radicals by donating an electron or a hydrogen atom [9]. Moreover, phenolic compounds can increase the activity of endogen antioxidant enzymes such as glutathione peroxidase, superoxide dismutase and catalase, and inhibit the activity of enzymes such as xanthinoxidase.

In 2020, H. B. Rashmi et al. published a detailed paper describing the properties of phenolic compounds related to their bioavailability and benefits for human health, as well as aspects connected to their stability during processing and storage [10].

**Figure 1:**
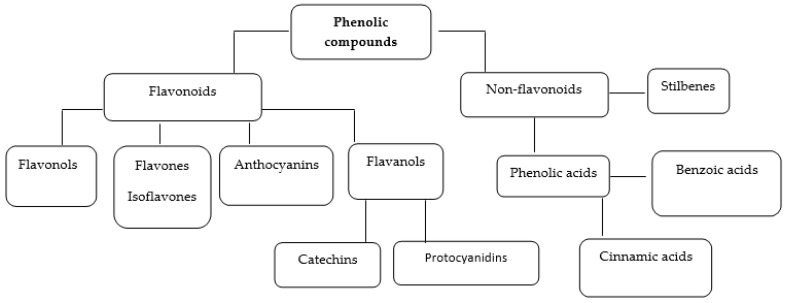
The main classes and subclasses of phenolic compounds.

From a structural point of view, phenolic compounds contain one or more aromatic rings substituted with hydroxyl groups, covering a wide variety of chemical structures, from simple molecules to polymeric compounds [11]. Figure 1 shows the scheme of the main classes of phenolic compounds.

Benzoic acids and cinnamic acids belong to the class of phenolic compounds. The chemical structures of phenolic acids are presented in Table 1.

Benzoic acids represent a class of phenolic compounds which contain seven carbon atoms (C1–C6) and which are present in foods, being frequent components of a complex structure, associated with hydrolysable tannins, sugars or lignin [12]. Derivatives of benzoic acid are produced through methoxylation or through the hydrolyzation of hydro benzoic acids. This category includes: benzoic acid, p-hydroxybenzoic acid, vanillic acid, gallic acid, syringic acid, veratric acid and salicylic acid [13].

Cinnamic acids are aromatic compounds with a side-chain of three carbon atoms (C3–C6), having ten carbon atoms in its structure. These participate in enzymatic oxidation reactions [14] and have, in turn, a series of compounds, including: cinnamic acid, o-coumaric acid, m-coumaric acid, p-coumaric acid, ferulic acid and caffeic acid. By cycling the lateral chain of the coumaric acid, the cinnamic acids can give other compounds, such as coumarins [15].

Many researchers have investigated this class of phenolic compounds present in a wide range of foods, from extraction and separation, to their identification and quantification. However, the study of compounds has encountered certain problems due to the high number of substances involved (thousands of compounds) and to their high polarity, compounds being very reactive and sensitive to enzyme action [16]. Among phenolic compounds, chlorogenic acid is of great interest because it has beneficial properties and the detection of this compound from natural samples is challenging.

## 2. General Information Regarding Chlorogenic Acid

Chlorogenic acids (CGAs) are natural compounds, predominant in superior plants. They represent a family of esters, formed between the quinic acid and certain trans-cinnamic acids. A series of conjugated structures—such as caffeoylquinic acid, di-caffeoylquinic acid, feruloylquinic acid and p-coumaroylquinic acid—exists, in several isomeric forms, in coffee beans [17].

CGA, (1S,3R,4R,5R)-3-{[(2E)-3-(3,4-Dihydroxyphenyl)prop-2-enoyl]oxy}-1,4,5-trihydroxycyclohexane-1-carboxylic acid, is the most abundant isomer found in nature and the most identified and quantified in natural samples.

**Figure 2:**
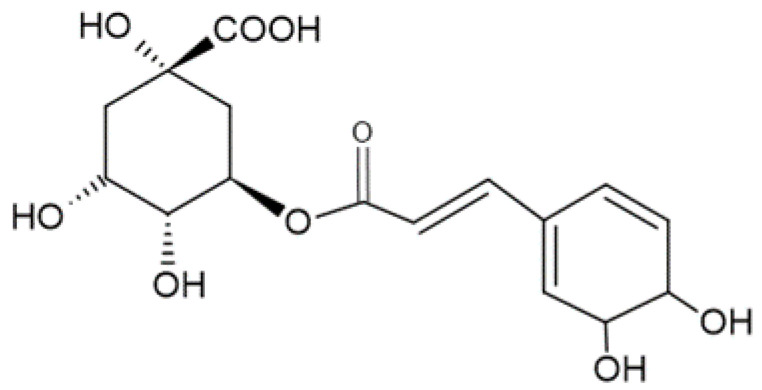
Chemical structure of chlorogenic acid.

The chemical structure of CGA is presented in Figure 2.

CGA was studied on a wide scale due to the fact that it is one of the main phenolic compounds of the human diet, with multiple benefits for human health. The compound is found in foods and aromatic plants such as: apples [18], artichokes [19], carrots [20], coffee beans [21], potatoes [22], grapes [23], tobacco leaves [24], tea [23], wormwood [25] and honeysuckle [26].

In recent years, a series of benefits for human health has been associated with CGA consumption. From among these, the following are noteworthy:

- Modulates the glucose metabolism in humans; therefore, CGA has an antidiabetic effect [27] on type 2 sugar diabetes, improving the quality of insulin;
- Prevents the development of cataracts as a result of its antidiabetic effect; this aspect has been indicated by the findings of studies carried out on laboratory animals;
- Reduces the relative risk of cardiovascular diseases, improving human vasoreactivity; moreover, it has antihypertension properties [28];
- Reduces the risk of biliary calculi production.

In laboratory tests, it has been shown that CGA has antioxidant properties, preventing cell deterioration [29]. Nevertheless, scientific evidence of preventive effects against chronic diseases remains unsubstantial, since CGA decomposes very rapidly in the human organism.

Moreover, CGA has anti-inflammatory [29] and anti-neurodegenerative [30] properties, contributing to the decrease of Alzheimer’s onset. In addition, most of the research carried out in relation to CGA’s benefits for human health focused on metabolic syndrome disorders, with the metabolic syndrome defined as the sum of physiological, metabolic, biochemical and clinical interrelated factors, which increase the risk of cardiovascular disease, type 2 sugar diabetes and, implicitly, of mortality due to various causes [31].

A study achieved in 2011 evaluated the antibacterial activity and the action mechanism of CGA against bacteria. The data on the minimum inhibition concentration (MIC) values showed that CGA had efficiently inhibited the increase of all the bacterial pathogen agents tested, and the MIC values ranged from 20 to 80 μg/mL. A study on the action of CGA against the pathogen agent indicated that this compound increased significantly the exterior and the permeability of the plasmatic membrane, the result being the loss of the barrier function. In short, CGA killed the strains of pathogen bacteria through causing irreversible modifications of the cellular membrane permeability [32].

### Pharmacokinetics of CGA

**Figure 3:**
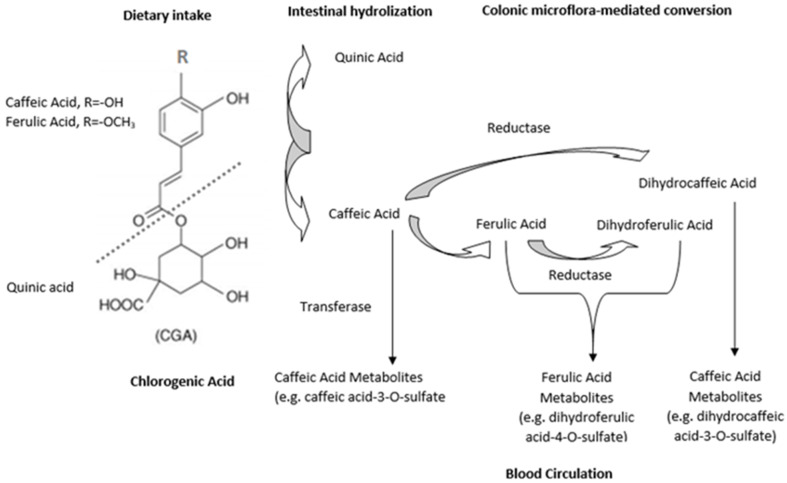
The major metabolic pathway of CGAs.

In a different experimental model regarding humans and animals, CGA and its metabolites were noticeable in the blood circulation system [33]. The main metabolites of CGA were ferulic, isoferulic and caffeic acid, which has been examined in the systemic circulation after regular consumption of coffee or green coffee extract. One-third of ingested CGAs contained in beverages and foods are physiologically absorbed in the small intestine. This can be measured chromatographically in the forms of 5-CQA, 4-CQA and 3-CQA in plasma by high-performance liquid [34,35]. The remaining two-thirds reach the large intestine where phenolic acid is further metabolized by the gastrointestinal microflora and then absorbed. The small intestine is the site where cleavage of quinic acid from FQA (feruloyl quinic acid) and CQA and then the release of ferulic acid and CA biochemically occurs. Furthermore, the colon plays a significant role in the conversion of both caffeic and ferulic acid to dihydroferulic acid and a major role in its absorption (Figure 3) [36].

Given that CGA is a compound of great interest, with many health benefits, developing reliable, simple, rapid and sensitive methods for its quantification is of major importance. Until now, various methods used in determining CGA have been described—from classical analytical determining methods to electrochemical methods involving the use of electrochemical sensors and biosensors.

## 3. Analytical Methods Used in Determining CGA

### 3.1. Instrumental Methods

Numerous analytical instrumental methods for determining CGA have been developed so far. Worth mentioning are the following: high performance liquid chromatography (HPLC), infrared spectroscopy, capillary and chemiluminescent electrophoresis.

#### 3.1.1. HPLC

HPLC is a separation method used in analytical chemistry to separate, identify and quantify chemical compounds. It is based on the different distribution of the components of a mixture between two phases: the stationary phase and the mobile phase [37].

Using this method, two collaborators, W. Liao and Y. Rui, determined CGA in the *Lonicerae flos* plant, the method’s principle involving the separation of this compound. The detection wavelength was set to 327 nm so as to determine the CGA content. Linearity was obtained in the range 8.8–89 µg/mL (r = 0.9999). Its average analytical recovery was 98.1%, and the relative standard deviation (RSD) was 1.85% (*n* = 51). The precision of the method was 0.82% (*n* = 5) [38].

Another study was carried out in 2012 and involved establishing a content determination method for CGA and 2-O-rhamnoside vitexin from *Crataegi fructus* extract through HPLC. Good values of linearity were obtained for both compounds, in the 1.34–164.80 and 1.18–148.7 mg·L^−1^ ranges. The analytical recoveries were 100.4% and 98.8%, while RSD were 1.5% and 1.3%, respectively [39].

L. Borisova-Jan et al. developed, in 2017, a HPLC based method of quantifying the derivatives of the caffeoylquinic acid in *Hieracium pilosella* L. The content of derivatives in the caffeoylquinic acid was determined and expressed as CGA. Validation showed that the chromatographic method has good selectivity, without interference peaks. Sensitivity, precision and repeatability proved adequate. The linearity of the sensor’s response to the compounds analyzed was between 1.5 and 150 µg/mL, corresponding to 0.03, up to 3% (g/w) [40].

Furthermore, in 2018, an analytical method based on separation with the aid of HPLC—with diode group detection for synchronized determination of CGA and five flavonoids (rutin, isoquercetin, quercetin, quercitrin, luteolin) in mulberry leaves (*Morus* sp.)—was developed and validated. In optimum working conditions, the extraction of CGA and of flavonoid compounds was achieved, with average analytical recovery values between 97.78% and 103.24%. The developed method was validated in relation to linearity, analytical recovery, precision and stability [41].

Another chromatographic method used for determining the CGA concentration in green coffee was liquid chromatography in reverse phase. It was described by a group of researchers in 2020. The identification was carried out through comparing the retention time of the pure analytical standard with the retention time of the CGA in the analyzed samples. An excellent linearity was obtained in the 12.33–143.50 µg/mL range. The value of the detection limit was 0.29 pg, and that of the quantification limit was 0.96 pg. The obtained results demonstrated that the used method was characterized by a wide linearity range, and that it was precise, with analytical recoveries between 97.87 and 106.67% [42].

#### 3.1.2. Near Infrared Spectroscopy (NIRS)

NIRS was proposed, in 2015, as a rapid and non-destructive method for the evaluating content in relation of three compounds (caffeic acid, catechin and CGA) and three methylxanthines (caffeine, theophylline and theobromine) in samples of ground coffee from various producers. Good linearity ranges were obtained, with determination coefficients of 0.95, 0.92, 0.88, 0.71 and 0.84 for caffeine, caffeic acid, catechin, chlorogenic acid and theophylline [43].

The feasibility of the NIR method for determining CGA, luteolin and 3,5-O-di-caffeoylquinic acid in species of *Chrysanthemum* was analyzed in a study published in 2019. The values of the prediction correlation coefficient (r_p_^2^) were: 0.924, 0.927, 0.933 for CGA, luteolin and 3,5-O-di-caffeoylquinic acid. The results indicated that NIR spectroscopy, combined with multivarious calibration methods, could be considered a useful tool in the rapid determination of biological compounds active in *Chrysanthemum species* [44].

A recent study (2021) was based on Fourier transform near infrared spectroscopy (FT-NIR). This method is used to determine the compounds in the samples and to classify them. This study developed a rapid, simultaneous, non-destructive method of analysis for eight compounds in the *Flos Mume* plant. The results indicated that only the neochlorogenic acid, CGA, rutin, hyperoside (quercetin-3-O-galactoside) and isoquercetin had concentration values which could be determined, the CGA content being the largest. The other components were excluded due to low concentrations. Through this study, a distinction among the various types of *Flos Mume* could be made, based on the CGA content, which shows that CGA has the potential to become a key indicator for use in quality evaluation [45].

#### 3.1.3. Capillary Electrophoresis

The technique of local capillary electrophoresis, combined with the assisted extraction of microwaves, was developed for the very first time in 2010 by Z. Li et al. to rapidly quantify CGA in tobacco residues. Being a new method, this procedure was optimized, validated and compared to conventional methods, namely extraction by ultrasound and extraction through reflux. The obtained results demonstrated that the technique of local capillary electrophoresis, combined with the assisted extraction of microwaves, has good linearity (R^2^ is 0.991, 0.003–0.5 mg/mL), detection limit (0.003 mg/mL) and quantification limit (0.01 mg/mL), as well as adequate precision, with an RSD value of 4.28% [46].

Another study, carried out in 2012, described a new method of infrared assisted extraction, doubled by electrophoresis, used to determine CGA in a traditional Chinese medicine, namely honeysuckle. The effects of pH and the concentration of the buffer solution, the tension applied for separation, the injection time, the IR irradiation time and the concentration of the standard sample in the extraction solution were all analyzed. Good linearity (r^2^ > 0.9996) in the studied concentration range was observed, and the stability of the solutions was found to be high. The CGA analytical recoveries were 95.53% up to 106.62%, and the relative standard deviation was under 4.1% [47].

A rapid and safe method based on capillary electrophoresis to simultaneously determine twenty polyphenolic compounds in less than 27 min was described, in 2015, by F. Gatea et al. It was assumed that these compounds are found in propolis and plant extracts, CGA being one of them. The linearity ranges used to quantify the compounds was satisfactory, with correlation coefficients between 0.997 and 0.999 for all the twenty compounds analyzed. The method showed good performance characteristics, namely detection and quantification limits from 0.02 to 1.75 and from 0.07 to 5.77 µg·mL^−1^, respectively. The values of the relative standard deviation for repeatability did not go above 4.86% for intra-daily tests and 5.07% for inter-daily tests. The results of the analytical recovery tests were between 87.4% and 114.2% for the *Origanum* sample, and between 85.0% and 111.0% for the propolis sample [48].

#### 3.1.4. Chemiluminescence

In 2007, Wang et al. described a method for detecting CGA based on the chemiluminescent reaction of potassium permanganate with CGA in the presence of formaldehyde as potentiator. This indicated a difference in the intensity of potassium permanganate chemiluminescence and formaldehyde in the presence of CGA, as compared to that in the absence of CGA, for a concentration range from 5.0 × 10^−8^ to 5.0 × 10^−5^ g/mL. The detection limit obtained was 5.7 × 10^−9^ g/mL when the collection rate was 150 injections per hour. The method was applied successfully to determine CGA in fruit, with analytical recoveries of 100 ± 6% [49].

In 2020, a rapid and simple method was developed to determine various phenolic acids (ferulic acid, CGA and salicylic acid) in plants through capillary electrophoresis with chemiluminescent detection. The simple chemiluminescent detection was carried out in a temperature-controlled laboratory constructed interface, through using potassium permanganate as chemiluminescent reactive. The detection limits for ferulic acid, CGA and salicylic acid were 35, 58 and 37 µg/mL, and the quantification limits were 117, 193 and 123 µg/mL. The proposed method was applied successfully in the quantitative determination of the phenolic acids in *Lonicera japonicaflos* and *Rhizoma Chuanxiong* [50].

Among these reported methods, near infrared spectroscopy (NIR) needs expensive equipment and calibrations, which involves supplementary costs in carrying out the determinations. The analyses through capillary electrophoresis have the disadvantage of not being very sensitive. The chromatographic methods need large quantities of organic solvents, expensive and laborious equipment [51]. By comparison, the UV-Vis method has the advantage of being simple, cheap and rapid in determining CGA in coffee beans. Nevertheless, a direct spectral determination of coffee beans is difficult to carry out due to the spectral overlap with caffeine [52].

Although these instrumental methods have proven to be efficient in quantifying CGA and its derivatives, a series of disadvantages has also been noted: they are time consuming and laborious, necessitating expensive equipment to carry out analyses [53].

### 3.2. Electrochemical Methods

In recent years, electrochemical methods have been investigated on a wide scale in view of determining phenolic compounds, the main reasons being their simplicity, high sensitivity, rapid response and reduced cost [54]. In addition to their use in the fundamental processes of oxidation and reduction to discover reaction mechanisms, these techniques are also used in studying the kinetics and thermodynamics of ion and electron transfer processes [55].

Electrochemical methods represent, most likely, one of the most adequate options in connection with phenolic compounds. The largest part of the biological activity of these compounds is due to their capacity to donate electrons to a wide range of receiving species. The redox potential of natural phenolic compounds covers a significant variety, which represents the first source of selection allowing the selective analysis of various electroactive compounds through various voltammetric techniques such as cyclic voltammetry (CV), differential pulse voltammetry (DPV) or square wave voltammetry (SWV) [56].

Electroanalytical methods were developed in view of determining CGA, even using metallic electrodes or carbon-based electrodes, without attempts at increasing selectivity through specific detection elements, but with the aim of improving sensitivity by coating with nanostructured materials, particularly graphene, carbon nanotubes or conducting polymers [57].

**Figure 4:**
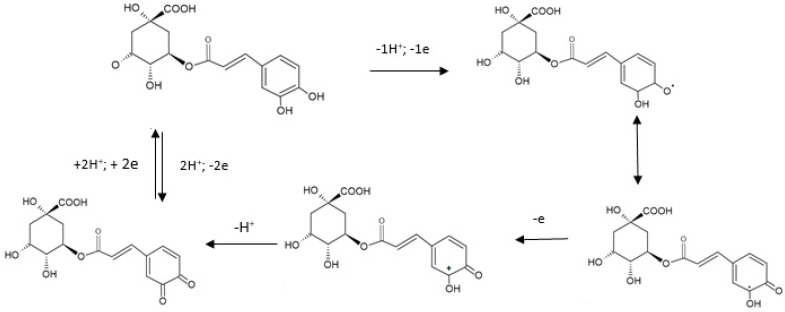
Electrochemical oxidation mechanism of CGA [58].

The electrochemical electrooxidation mechanism of CGA involves, in a first stage, the pre-dissociation of a proton with the formation of a monoanionic species. This, in turn, is oxidated to generate a radical. The radical formed is prone to a new rapid electronic transfer with the formation of a carbocation which, through dehydration, forms the adequate quinone. During reverse scanning, the quinone formed is reduced to CGA. This oxidation mechanism is presented in Figure 4 [58].

#### 3.2.1. Electrochemical Sensors Used in Determining CGA

Through the years, several studies on establishing CGA with the aid of various electrochemical sensors based on modified electrolytes have been carried out.

A study carried out in 2011 focused on the construction of a molecularly imprinted electrochemical sensor for the selective detection of CGA through adding a molecularly imprinted siloxane film prepared through the sol-gel process onto the surface of the gold electrode. The surface of the molecularly imprinted siloxane film was electrochemically characterized using differential pulse voltammetry (DPV). The sensor obtained was tested both in a solution containing CGA and in solutions of other similar molecules, with an excellent selectivity towards CGA. The current of the anodic spot was linear in the 5.0 × 10^−7^–1.2 × 10^−5^ mol/L range. The detection limit was also calculated, with a value of 1.48 × 10^−7^ mol/L being obtained. To evaluate how applicable the sensor is, it was used to determine the CGA concentration in coffee and tea samples, with analytical recovery values between 94.3% and 107.9% being obtained, which suggests that the method has good precision and high potential in analyzing real samples [59].

**Figure 5:**
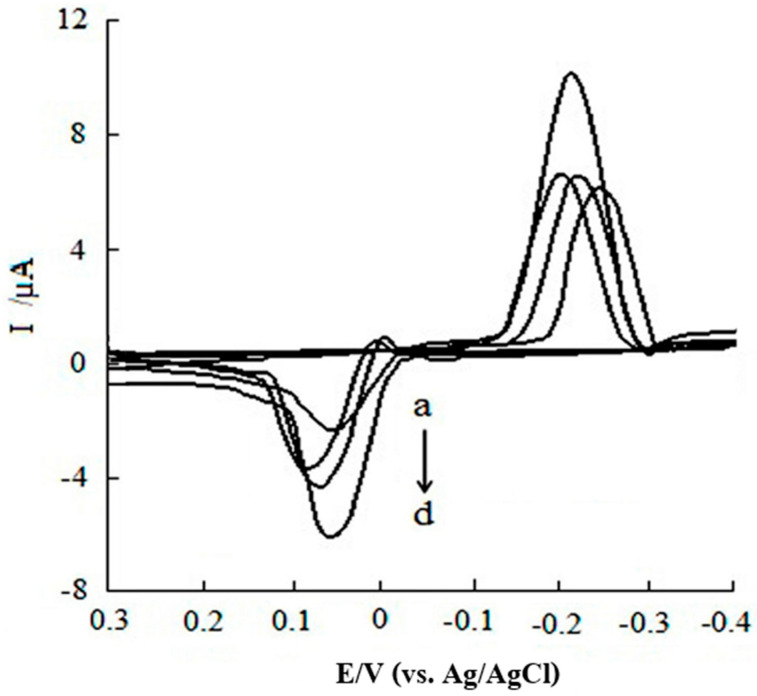
Influence of different supporting electrolytes on peak current: (a) 0.10 mol/L potassium hydrogen phosphate–potassium dihydrogen phosphate buffer; (b) 0.10 mol/L phosphate buffer solutions; (c) 0.10 mol/L citric acid buffer; (d) 0.10 mol/L acetic acid–sodium acetate buffer [52].

In another study, carried out in 2016 by Ma. Xiaoyan et al., CGA was determined using a screen-printed carbon electrode with multi-walled carbon nanotubes (MWCNTs/SPE). Two detection techniques were used, namely: cyclic voltammetry (CV) and DPV. The influence of four supporting electrolytes on the sensor response was evaluated, as shown in Figure 5. A pair of redox peaks was highlighted in the case of all the support electrolytes used, but the highest peak was obtained for the 0.1 mol/L acetic acid–sodium acetate buffer solution.

In optimum conditions, the method indicated linear ranges from 0.17 to 15.8 µg/mL, a limit of detection of 0.12 µg/mL being obtained. The sensor was later used in the quantitative determination of CGA in coffee beans and green tea, a relative standard deviation (RSD) between 1.33% and 4.77% and analytical recoveries of 94.74–106.65% being obtained. The results achieved were compared to those of the HPLC method, a good correlation being noted [52].

Another study on the determination of CGA was based on the synthesis of a new compound, namely a Fe_3_O_4_ superparamagnetic nucleus encapsulated in an organic metal framework shell, Fe_3_O_4_@MIL100(Fe), through the layer-to-layer method. The thickness of the shell could be controlled through auto-assembly cycles between FeCl_3_·6H_2_O and 1,3,5–benzenetricarboxylic acid. The characterization of the composite materials was performed through X-ray diffraction, transmission electronic microscopy (TEM), Fourier transform infrared spectroscopy and N_2_ adsorption/desorption isotherms. This material was used to obtain a new sensor to determine CGA. In optimum conditions, the electrochemical sensor was shown to quantitatively detect CGA in the 0.1–10.0 μmol·L^−1^ and 10.0–460 μmol·L^−1^ range. The detection limit was 0.05 μmol·L^−1^. Another aspect studied was the repeatability of the sensor through seven successive determinations of 20 μmol·L^−1^ CGA in PBS, with a relative standard deviation (RSD) of 4.2%. The stability of the sensor was evaluated through carrying out 30 cycles, which indicated that the catalytic properties of the modified electrode were preserved, without a significant decrease of the current intensity. Finally, in view of determining the practical applicability of the new sensor, a test on the CGA content of the real samples was carried out through the method of addition, using DPV. The values of the analytical recoveries varied from 96.0% to 103.8%, which shows that the new sensor has good precision and can be successfully used in analyzing real samples [60].

Another study carried out in 2016 was aimed to prepare a sensor based on metal-organic/titanium dioxide (UiO-66-NH_2_/TiO_2_) nanocomposite structures so as to determine CGA. The nanocomposite was prepared through a simple hydrothermal reaction, the electrocatalytic activity of the compound for CGA oxidation being due to the synergic effect of UiO-66-NH_2_ and TiO_2_.

**Figure 6:**
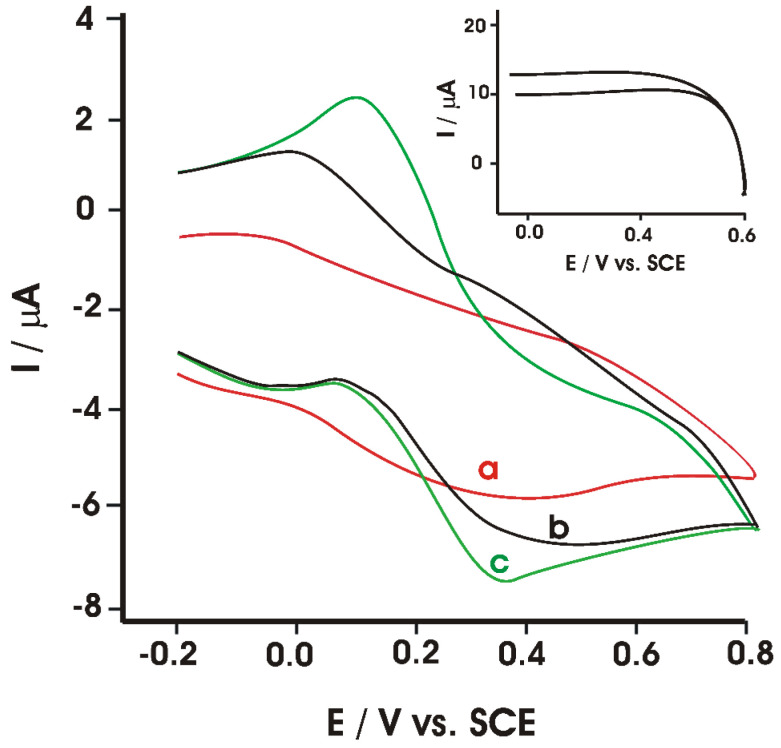
Cyclic voltammograms of chlorogenic acid at the bare GCE (a), UiO-66-NH_2_/GCE (b), and UiO-66-NH_2_/TiO_2_/GCE (c) in 0.1 mol L^−1^ pH 6.0 PBS in the presence of chlorogenic acid. Scan rates: 100 mV·s^−1^. Inset: Cyclic voltammograms of the UiO-66-NH_2_/TiO_2_/GCE in 0.1 mol L^−1^ PBS (pH 6.0) in the absence of chlorogenic acid. Adapted from [61].

The electrochemical effect of CGA was studied at pH 6.0, using a phosphate buffer solution, through CV. In a PBS solution, the appearance of redox peaks cannot be noticed, which indicates the fact that the UiO-66-NH_2_/TiO_2_ nanocomposite is not electroactive in the potential range selected. On adding CGA to PBS, a pair of redox peaks appears, which are attributed to the electrochemical behavior of CGA (Figure 6).

Cyclic voltammograms of the sensor immersed in 10.0 μmol·L^−1^ CGA solution, at various scan rates, were also achieved. It was noticed that the current intensity is directly proportional with the square root of the scan rate, which suggests that the CGA redox process is influenced by the diffusion of the electroactive species on the electrode surface.

**Figure 7:**
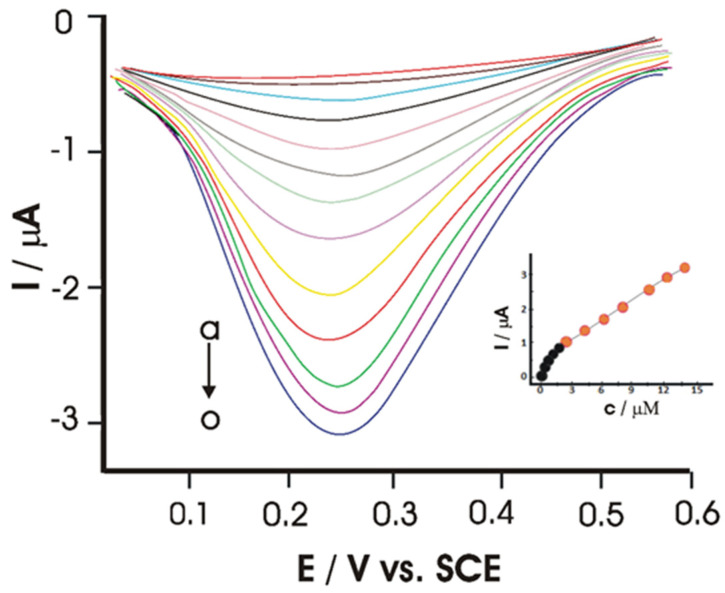
Differential pulse voltammograms at UiO-66-NH_2_/TiO_2_/GCE for different concentrations of chlorogenic acid in pH 6.0 PBS (from a to o) 0.01, 0.03, 0.05, 0.07, 0.1, 0.3, 0.5, 0.7, 1.0, 3.0, 5.0, 7.0, 10, 13 and 15 µmol·L^−1^. The inset shows the relationship between the peak current and the chlorogenic acid concentration. Adapted from [61].

The variation of current intensity based on CGA concentration was studied next on the UiO-66-NH_2_/TiO_2_ surface, using DPV, as shown in Figure 7. From the results obtained, LOD was calculated, its value being 7 nmol·L^−1^. The relative standard deviation of 3.67% for 9 measurements of 1.0 μmol·L^−1^ CGA indicates the good repeatability of the analyzed sensor.

Finally, to confirm the performance of the proposed method in real samples, applicability was tested in coffee and tea samples. Good recoveries were obtained for all the samples, in the 96.0–102.0% range, suggesting that the proposed method is efficient and has high precision [61].

In 2007, N. Mohammadi et al. carried out a study which consisted in obtaining a modified carbon paste electrode, based on defective mesoporous carbon (DMC) and an ionic liquid (1-butyl-3-methylimidazolium-hexafluorophosphate-BMIM.PF_6_) at room temperature, used in the voltametric determination of CGA in plant extracts. DMC was synthesized through a simple method, using nanosilicon as hard template, sucrose as carbon source, and KNO_3_ as flaw producing agent. The characteristics of the modified electrode (IL/DMC/PE) were evaluated through CV, electrochemical impedance spectroscopy and chronocoulometry. The proposed sensor showed remarkable efficiency related to the electrochemical reaction of CGA in aqueous solutions. Moreover, the electrocatalytic behavior was studied through SWV, due to the better sensitivity and resolution of this method in comparison with those of CV.

**Figure 8:**
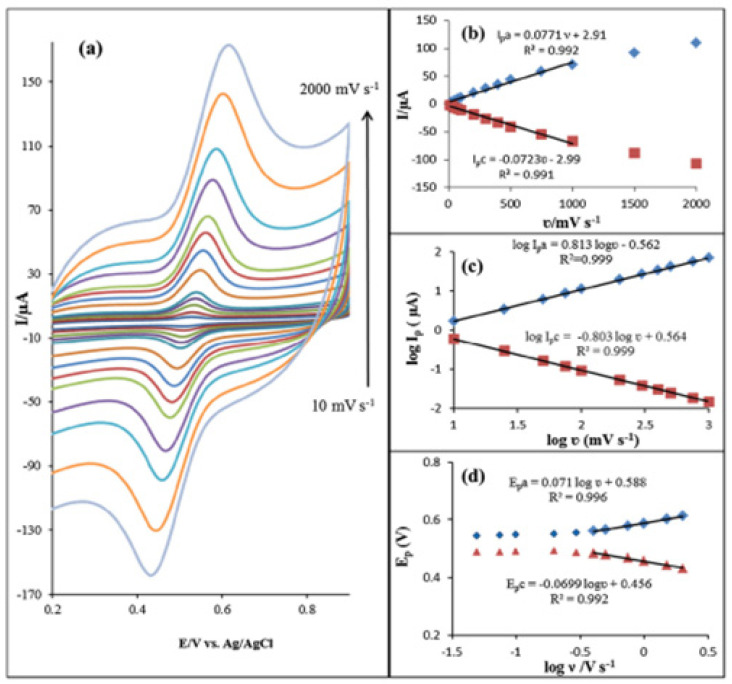
(**a**) CV responses of the modified electrode in Britton–Robinson buffer solution (pH 2) at scan rates (inner to outer) 10, 25, 50, 75, 100, 150, 200, 300, 400, 500, 750, 1000, 1500 and 2000 mV·s^−1^. (**b**) The plot of anodic and cathodic peak current vs. scan rate. (**c**) The plot of anodic and cathodic peak current vs. logarithm of scan rate and (**d**) the variation of anodic and cathodic potential vs. logarithm of scan rate. Published from [62] with the permission of the publisher.

Figure 8 shows the cyclic voltammograms obtained and the calibration curve for various concentrations of CGA. In optimum conditions, the currents of the anodic peak increased linearly with the CGA concentration, in the range 0.02–2.5 × 10^−6^ mol·L^−1^, the limit of detection obtained being 1 × 10^−8^ mol·L^−1^.

The relative standard deviation of five successive measurements with the same electrode toward 1 × 10^−6^ mol·L^−1^ CGA was approximately 3%, suggesting good repeatability of the studied sensor. The long-term stability of the modified electrode was also verified, the current intensities following the measurements made for 1 × 10^−6^ mol·L^−1^ CGA remaining at 95% of its initial value after a month of storage at room temperature.

The applicability of the sensor studied was evaluated by determining the CGA content in two plant extracts. The results obtained were satisfactory, with analytic recovery in the 98.6–105.8% range, which indicates that the proposed sensor could be efficiently applied in reliably determining CGA from plant extracts [62].

Another study was aimed to prepare the TAPB-DMTP-COFs composite doped with gold nanoparticles (TAPB, 1,3,5-tri(4-aminophenyl)benzene; DMTP, 2,5-dimethoxyterephaldehyde; COFs, covalent organic frameworks), then used to develop a new electrochemical sensor. This indicated a good electrocatalytic activity on CGA oxidation in phosphate buffer solution at pH 7.0. The electrochemical sensor obtained showed a linearity range between 1.00 × 10^−8^ and 4.00 × 10^−5^ M, with limit of detection 9.50 × 10^−9^ M. According to the method proposed, the sensor was applied for determining CGA from coffee samples, fruit juices and plant extracts, the analytical recovery being 99.20–102.50%. The results of CGA determination were according to those obtained through the HPLC method [63].

R. Chokkareddy et al. developed a new sensor (LGN-MWCNTs-CuONPs-GCE) to detect CGA in coffee samples, modifying the glassy carbon electrode with nanocomposites based on multi-walled carbon nanotubes (MWCNTs), copper oxide nanoparticles (CuONPs) and a polymer–lignin (LGN). Using CV, the quasi-reversible nature of the process was highlighted, as well as the fact that it is controlled by the adsorption of electroactive species on the surface of the sensor. Nanoparticles and synthesized nanocomposites were characterized through FTIR, transmission electronic microscopy (TEM) and X-ray diffraction analyses (XRD). DPV was applied and the anodic peak was used in the quantitative determination of CGA. The sensor proposed showed linear responses in the 5 µM and 50 µM range, the limit of detection (LOD) being 0.0125 µM, and the limit of quantification (LOQ) 0.2631 µM.

The LGN-MWCNTs-CuONPs-GCE sensor was then applied to detect CGA in coffee samples, with the analytical recoveries varying from 97 to 106%. Thus, the sensor developed was successfully used to analyze the CGA content in these samples. Moreover, the electrophile, nucleophile reactions of CGA and the studies on CGA docking were carried out to better understand the redox mechanisms; they were supported by calculations of the density-functional theory [64].

**Figure 9:**
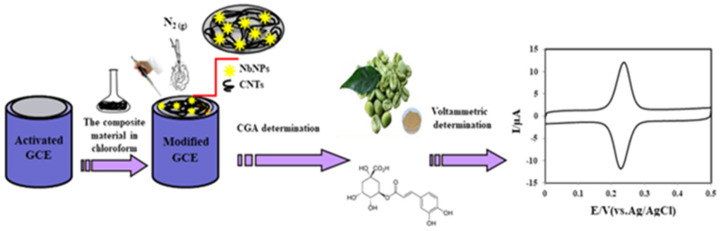
A simple illustration for the preparation of the platform and its application in electroanalysis. Published from [67] with the permission of the publisher.

Another study, carried out in 2020, aimed to develop a new voltammetric method for CGA detection using a glassy carbon electrode modified with niobium nanoparticles (NbNPs) and multi-walled carbon nanotubes. To characterize the electrode sensitive material, analytical techniques—such as energy dispersion X-ray analysis (EDX), scanning electronic microscopy (SEM) and X-ray diffraction (XRD) spectroscopy—were used. Figure 9 shows the scheme for achieving the sensor and for use in the voltammetric analysis of the samples.

Square wave voltammetry (SWV) was also used to determine the linear range and the limit of detection (LOD). What was observed was that the intensities of the peaks were linear with the CGA concentration, in the range 2.0 × 10^−9^ M and 2.0 × 10^−6^ M, with LOD 2.0 × 10^−9^ M. This value of the LOD obtained indicated that the voltammetric method proposed, using a NbNPs/CNTs/GCE sensor, was extremely sensitive to determine CGA as compared to other LOD values obtained with other electrodes [51,65,66].

**Figure 10:**
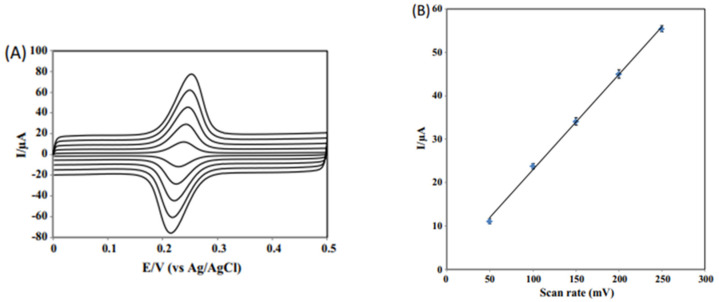
(**A**) CVs of 1.0 × 10^−7^ M CGA at NbNPs/CNTs/GCE in 0.1 M PBS at pH 7.0. Scan rate: 50 mV/s, 100 mV/s, 150 mV/s, 200 mV/s, 250 mV/s (bottom to top). (**B**) A plot of anodic peak currents of CGA versus scan rates. Published from [67] with the permission of the publisher.

To highlight the possible mechanism of the redox process, CVs with different scan rates were used, the response obtained being linear with the scan rates, in the 50–250 mV/s range. This suggests that the process is controlled by the adsorption of the species, which are active on the surface of the electrode (Figure 10).

Subsequently, the sensor proposed was used to determine CGA in green coffee samples, in tomato juice and carbonated drinks. The quantity of CGA obtained was 19.1 mg/g in the coffee beans, 11.4 mg/100 mL for the tomato juice, and 1.84 mg/100 mL for the carbonated drinks. The analytical recoveries of 99% and 101.2% demonstrated that the method proposed is accurate for determining CGA in real samples [67].

Detecting CGA in clinical samples for studies on metabolic kinetics is a challenging mission due to low concentration and lack of sensitive analytical methods. Taking this analytical issue into account, a porous pencil lead electrode (PLE) was used to detect CGA, using SWV as electroanalytical method. Sensitivity was significantly increased due to CGA accumulation in the porous structure of the electrode. In optimum conditions, CGA concentration was linear in the range 7.7 × 10^−8^–7.7 × 10^−6^ M, with limit of detection 4.5 × 10^−9^ M. Later, the electrode was used to determine CGA in human urine, with analytical recoveries between 89.6% and 105.5%. This highlighted the precision of the method, recommended for use in the rapid and sensitive detection of CGA in human urine samples [68].

In a recent study, three screen-printed electrodes—namely the screen-printed carbon electrode (C-SPE), the screen-printed carbon electrode modified with graphene (GPH-SPE), and the screen-printed carbon electrode modified with graphene and gold nanoparticles (GPH-GNP-SPE)—were used to study the electrochemical behavior of CGA in watery solutions, the method of detection employed being CV.

**Figure 11:**
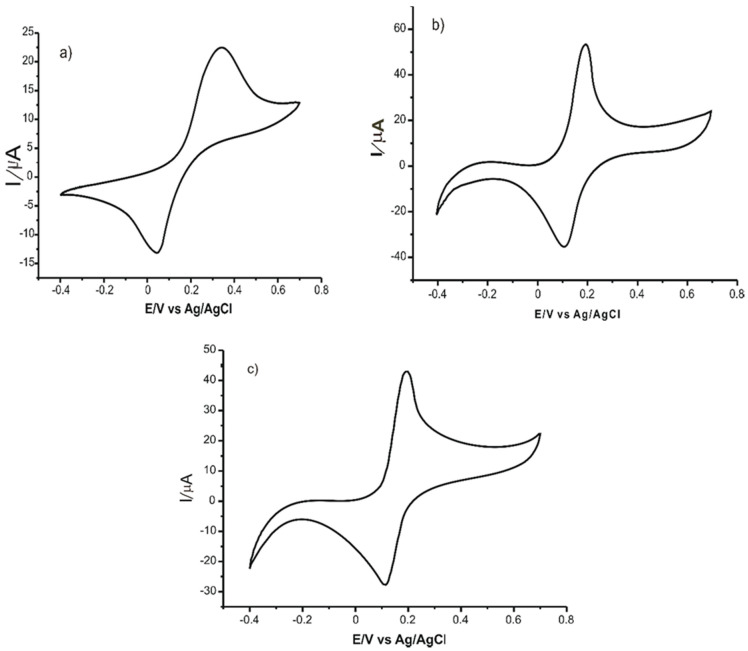
CVs of: (**a**) C-SPEs, (**b**) GPH-SPE and (**c**) GPH-GNP-SPE immersed in 10^−3^ M CGA solution (support electrolyte 10^−1^ M PBS solution). Scan rate 0.1 V·s^−1^ [58].

Cyclic voltammograms of the 10^−3^ mol/L CGA solution were recorded. The 10^−1^ M (pH = 7.0) phosphate buffer solution was used as a supporting electrolyte. To obtain a stable response of the sensor, five cycles were necessary in the optimized potential range (from −0.4 V to 0.7 V). The cyclic voltammograms presented in Figure 11 were obtained after signal stabilization.

Further, the limits of detection and the limits of quantification were determined for all three electrodes studied, and the values obtained are shown in Table 2.

Low values of the limit of detection and of the limit of quantification were due to the sensors used in the study, which demonstrated the feasibility of the voltammetric method for analyzing CGA in real samples such as nutraceutical products. Thus, the next stage of the study involved the quantitative determination of CGA in three nutraceutical products. Two methods were used: CV for determination and FTIR (spectrometric method) for validation of the voltammetric method’s accuracy. The results obtained were very close to those obtained through the standard analysis method, and the confidence level was 99% [58].

The electrochemical sensors achieved and used to analyze CGA were based on nanomaterials, conductive organic polymers and nanocomposites based on carbon structures decorated with semiconductive particles (gold nanoparticles, copper oxide nanoparticles, niobium nanoparticles). They were all used successfully in the sensitive and precise analysis of CGA, especially in vegetable products.

However, some facts reduce the applicability of the sensors in CGA analysis. On one hand, the inactivation of the electrode active surface area due to the adsorption of macromolecules or of reaction products greatly affect the electrode response stability. Moreover, coexisting components, which may be present in concentrations much larger than the analytes, may severely interfere in the determination of the targeted compound. Complicated sample pretreatments are often employed to eliminate or separate interfering components.

These disadvantages can often be controlled by changing the surface of the sensors using mediators and/or enzymes to improve quantitative measurements and also to adapt the electrode to specific responses to certain application. Electrochemical biosensors represent very interesting devices for on-line monitoring beside single-detection uses. The analytical performances of the electrochemical biosensors in CGA analysis, as well as their key fabrication details, are reported in the next section.

#### 3.2.2. Electrochemical Biosensors Used in CGA Detection

At present, a variety of enzymes belonging to the oxidoreductase class (tyrosine [69], laccase [70] and peroxidase [71]) are used in the electrochemical detection of phenolic compounds and in evaluating their antioxidant activity through biochemical oxidation followed by electrochemical reduction [72].

These enzymes may be considered functional components of the electrochemical sensors. However, the substrate recognition process is based on their mechanism of action. Consequently, they also play the role of recognition elements for target cells, and the substrate selectivity leads to the detection of specific classes of phenolic compounds or other chemical compounds. Their use on the electrode’s surface is not a novelty, but necessitates solving certain typical issues which result from the obligation of correctly immobilizing the enzyme, from the compatibility with more types of materials and from the modifications of the conductive properties of the active surface [73].

**Figure 12:**
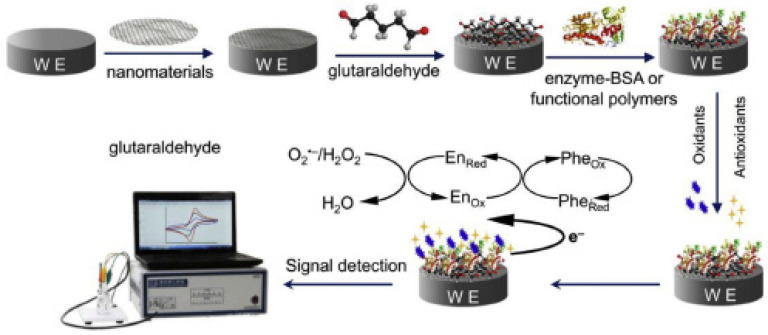
Schematic illustration of the preparation of modified enzyme-based electrode. Published from [74] with the permission of the publisher.

The schematic representation of preparing a biosensor using the enzyme is shown in Figure 12 [74].

A study carried out in 2008 described the development of two biosensors for determining CGA through immobilizing homogenized fresh bean sprout (*Vigna radiata*) in chitosan microspheres (biosensor I) and silicon microspheres (biosensor II), crosslinked with glutaraldehyde and epichlorohydrin. The bean sprout tissue acts like a source of peroxidase which, in the presence of hydrogen peroxide, catalyzes the oxidation of CGA. The CGA concentration was linear in the range 4.89 × 10^−6^–3.20 × 10^−4^ for biosensor I, and in the range 4.89 × 10^−6^–4.85 × 10^−5^ for biosensor II. The limits of detection obtained for the two biosensors were 8.02 × 10^−7^ M for biosensor I and 8.52 × 10^−7^ M for biosensor II. Last but not least, the two biosensors demonstrated good long-term stability (4 months, 300 determinations) and reproducibility, with a relative standard deviation of 3.5% for biosensor I and 1.5% for biosensor II. The CGA recoveries from four coffee samples varied between 96.5% and 102.6% (biosensor I), and between 91.3% and 115.5% (biosensor II). Thus, the results obtained for determining CGA in real samples using the proposed biosensors and those obtained through using the capillary electrophoresis method were in accordance, at a confidence level of 95% [75].

One year later, in 2009, a study on constructing a biosensor based on ionic liquid, 1-n-butil-3-methylimidazole hexafluorophosphate containing iridium dispersed nanoparticles (Ir-BMI.PF_6_) and polyphenol oxidase (PPO) was carried out. This enzyme was obtained from sugar apple (*Annona squamosa*), immobilized in crosslinked ionic chitosan with oxalate. The biosensor obtained was used to determine CGA through square wave voltammetry.

**Figure 13:**
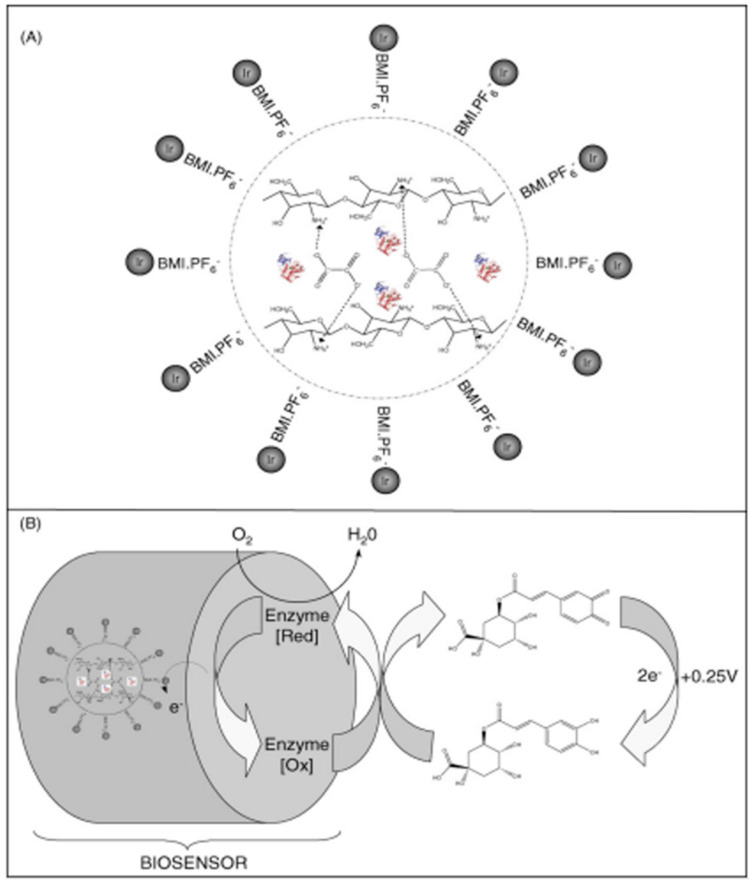
Schematic representation of (**A**) immobilization of PPO on chitosan crosslinked with oxalate and (**B**) reaction involving chlorogenic acid on the surface of the biosensor. Published from [76] with the permission of the publisher.

The positive charged amino groups of chitosan can react with the negative charged oxalate through electrostatic attraction to form ionically crosslinked networks. Figure 13 shows the schematic representation of immobilizing PPO in the vegetal material (sugar apple) in the ionically crosslinked chitosan, obtaining the proposed biosensor with high stability and durability. Polyphenol oxidase catalyzes CGA oxidation at the corresponding o-quinone, which is electrochemically reduced, the same compound being obtained at +0.25 V vs. Ag/AgCl. In these experimental conditions, the response of biosensor was linear with the CGA concentration in the range 3.48 × 10^−6^ and 4.95 × 10^−5^ mol L^−1^, with a limit of detection of 9.15 × 10^−7^ mol L^−1^. Later, the biosensor was applied for CGA determination in decaffeinated organic coffee samples, and the results obtained were compared to those obtained by using the capillary electrophoresis method. The study on CGA recovery in these samples gave values between 93.2% and 105.7%. Therefore, immobilizing enzyme in biocompatible chitosan, together with Ir-BMI.PF_6_, applied to determine CGA in coffee beans, proved to be a simple and efficient analytical method [76].

Another study consisted in developing a network of sensors based on a double screen-printed carbon electrode to simultaneously determine CGA and caffeine. One of the working electrodes was modified with platinum nanoparticles, reduced graphene oxide and laccase (C-SPE/Pt-NPs/RGO/Lac-biosensor) to determine CGA, while the second working electrode was modified with reduced graphene oxide and Nafion (C-SPE/RGO/Nafion-sensor) and was used to determine caffeine. Cyclic voltammetry was the method used to characterize and optimize the sensor array, while chronoamperometry was used to study the bioelectrocatalytic response. The C-SPE/Pt-NPs/RGO/Lac biosensor, used to detect CGA, had a sensitivity of 0.02 µA/µM and a limit of detection of 2.67 µM, while the C-SPE/RGO/Nafion sensor, used to detect caffeine, had a sensitivity of 1.38 µA/µM and a limit of detection of 0.22 µM. The sensor array developed was later used to determine the two phenolic compounds in real coffee samples. It could be concluded that, due to its simplicity, feasibility and accessibility, the sensor array developed could represent the basis for a valuable analytical tool, capable of analyzing both the CGA content and the content of caffeine in coffee samples, thus providing important information about the phytochemical composition of samples [77].

Similar to the previous study, another paper described the development of amperometric biosensors based on glassy carbon electrodes modified with graphene oxide and MWCNTs. The two biosensors were achieved with the aid of two enzymes, namely tyrosine (Tyr) and laccase (Lac). Before the deposition of the enzyme, the graphene oxide was reduced through the electrochemical method, using CV. The immobilization of the enzyme on the surface of the modified electrode was optimized using various agents (bovine serum albumin and glutaraldehyde as reticulation agent, chitosan and Nafion). The conditions for manufacturing and storing biosensors were established to obtain a good enzyme retention, high sensitivities and durable devices.

**Figure 14:**
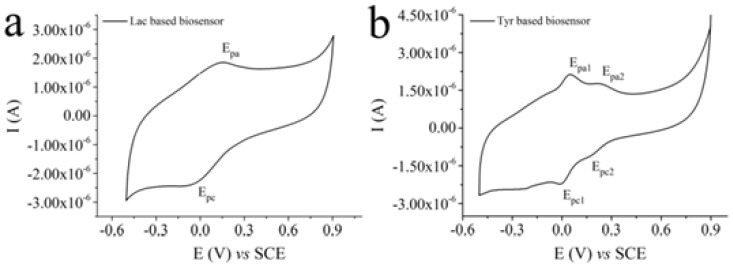
CV characterizations recorded in 0.1 M acetate buffer, pH 4.5 for a biosensor obtained by casting 10 µL of a suspension containing 8 mg·mL^−1^ Lac (**a**), and in 0.05 M PBS, pH 6.5, for a biosensor obtained by casting 10 µL of a suspension containing 6 mg·mL^−1^ Tyr, working under Ar atmosphere (**b**). Scan rate: 0.005 V·s^−1^. Published from [78] with the permission of the publisher.

Figure 14 shows the relevant electrochemical responses of the biosensors. It can be observed that, for the biosensor based on laccase, one pair of symmetrical redox peaks is highlighted, while for the biosensor based on tyrosine, two pairs of redox peaks are highlighted. This may be attributed to the carbon nanomaterial, which favors the transfer of electrons between the active centers of the tyrosine and the electrode.

The limit of detection calculated from the calibration curve was 0.3 µM. The biosensors thus obtained were used to determine the catechol of other polyphenols also, namely pyrogallol, epicatechin, gallic acid, 1,2-dihydroxibenzoic acid, caffeic acid, chlorogenic acid, rutin, catechin and dopamine. Finally, their practical applicability was demonstrated through estimating the total concentration of polyphenols in the juice sample, expressed as epicatechin equivalents [78].

The development of a new, ultrasensitive electrochemical biosensor to determine hydroquinone and CGA was described by F. Tulli et al. The biosensor was prepared through horseradish peroxidase immobilization (HRP) on nanohydrogels from *Laponite*, gold nanoparticle (AuNP) and copolymerized vinilbenziltrietilammonium polication with vinilbenziltiaminic groups. The structure and the active site of the enzyme were not modified on immobilization, which was determined through UV-Vis spectroscopy and FTIR. The biosensor showed remarkable electroanalytical properties for detecting CGA and hydroquinone, with a limit of detection of 1.6 ± 0.2 nM and 2.7 ± 0.1 nM, respectively. The biosensors were tested successfully to quantify the total content of polyphenols in green tea and yerba mate infusions and the results were comparable to those of the Folin–Ciocalteu method. The biosensor obtained had remarkable advantages due to its ultra-sensitivity, the small volumes of necessary samples and the short times needed for detection—all contributing to improving the analytical applicability [79].

**Figure 15:**
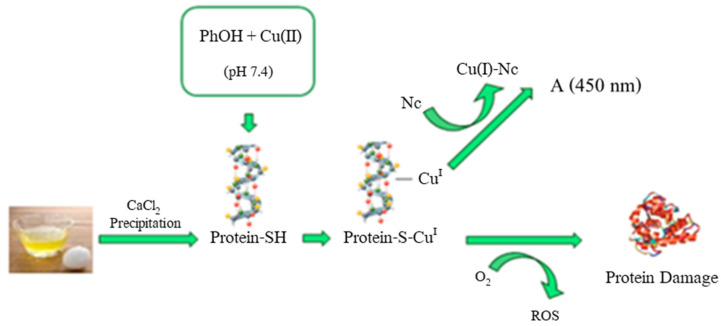
Schematic diagram of the proposed total pro-oxidant activity (TPA) assay using protein-based sensor. Reprinted with permission from [80]. Copyright 2021 American Chemical Society.

E. Akyuz et al. proposed the development of a biosensor based on proteins, which can measure pro-oxidant activities of phenolic compounds (Figure 15). To obtain the biosensor, proteins from egg white were precipitated with calcium chloride to obtain a complex of insoluble calcium proteins. This biosensor was used to determine the pro-oxidant activity induced by Cu(II) of antioxidants such as gallic acid, catechin, epicatechin, quercetin, CGA, myricetin and ascorbic acid. The analysis involved Cu(II) ion reduction at Cu(I) by the antioxidant compounds (which produce simultaneous reactive oxygen species), followed by linking the Cu(I) formed to the solid biosensor. Cu(I) linked to the protein is an indicator of the pro-oxidant activity of antioxidants on proteins, colorimetric determined at 450 nm with neocuproine (Nc). The method was applied to synthetic mixtures and plant infusions (sage, green tea, mint and rosemary). The advantages of this biosensor were related to the low costs, the possibilities of large-scale production, and use over a longer period of time [80].

The biosensors used to determine CGA were mainly based on enzymes (polyphenol oxidase, laccase, tyrosine and peroxidase) and on nanocomposite materials (platinum nanoparticles, gold nanoparticles, iridium nanoparticles), which facilitated the sensitive and selective detection from complex samples.

As in the case of sensors, enzymatic biosensors could have some drawbacks related to loss of enzymatic activity upon immobilization of enzyme and during the use of biosensor, as well as difficulties in recovering biosensor activity. These limitations can be avoided by using optimal enzyme immobilization techniques [81].

Enzyme immobilization on the surface of the electrodes is not a novel technique, but calls for the resolution of typical problems arising from the need of correct immobilization, compatibility with the advanced materials used in the development of the biosensor, changes in the conductive properties of the surface, and inactivation by inhibition at the catalytic site level [1].

The sensitive materials and the analytical features for the principal electrochemical sensors and biosensors used for CGA detection, most of them detailed above in this review, are summarized in Table 3.

## 4. Conclusions

The development of sensors and biosensors created a wide variety of new detection possibilities, as well as opportunities for the efficient use of electroanalysis. They have remarkable analytical properties and can become useful tools in clinical diagnosis due to the fact that their determinations are simple, selective, rapid and sensitive.

The present paper reviewed the most recent research carried out in view of determining CGA using electrochemical sensors and biosensors. Electrochemistry is the first choice taken into consideration for determining CGA due to the redox activity of this phenolic compound. Nevertheless, the method is only partially satisfactory with regard to selectivity in recognizing target analytes. In recent years, electroanalysis has undergone major innovations following the development of new, organic or inorganic, nanostructured materials, and of composite systems which combine the sensitive properties of both classes of nanomaterials. Such systems have been applied on a large scale in the analysis of CGA content in various natural origin samples, from green coffee beans, grapes and wine, to spices and vegetables.

Most electroanalytical detection methods which involve sensors and biosensors have very good selectivity and sensitivity in detecting CGA, without involving stages of preparation in relation to the samples to be analyzed. There are, however, certain limitations concerning biosensors, linked to the contamination of the active surface or the reduced enzymatic activity. These limitations could be decreased or avoided through using complex nanocomposite materials which are resistant to contamination and are capable of acting synergistically with the enzyme.

Future studies carried out will have to focus on methods of developing biosensors, which avoid active surface contamination and interferences produced by the presence of chemical substances in the sample analyzed, and which allow the possibility to simultaneously detect more analytes, having applicability in various fields. Optical sensors are largely still to be developed and molecular imprinting could still play a major role in this field to improve selectivity. Studying the detection of CGA adsorption, metabolization and excretion in the human organism is another important direction for inter- and multi-disciplinary research to be carried out in the years to come. A very important aspect that must be taken into account in this field is related to the complex composition of real samples, especially biological fluids, in which different compounds can interact, reducing redox activity and thus the electrochemical signal.

Another future development direction in electrochemical biosensitivity will be the development of implantable sensors for continuous health monitoring. For this purpose, new nanomaterials need to be integrated into specific devices so as to achieve good long-term stability and limit contamination. New detection techniques, such as electrochemical scanning microscopy, spectroelectrochemistry or ultra-fast cyclic voltammetry should be implemented for real-time CGA monitoring.

Moreover, the development of commercial systems for the analysis and determination of CGA based on sensors which imply a series of artificial receptors—peptides and aptamers—as well as the development of detection systems which are compatible with electrophoretic or chromatographic systems have to be taken into serious consideration in the near future.

## Figures and Tables

**Table 1:** Chemical structures of phenolic acids.

**Benzoic Acids**	**R_1_**	**R_2_**	**R_3_**	**R_4_**	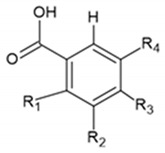
Benzoic acid	H	H	H	H	
p-Hydroxybenzoic acid	H	H	OH	H	
Vanillic acid	H	OCH_3_	OH	H	
Gallic acid	H	OH	OH	OH	
Syringic acid	H	OCH_3_	H	OCH_3_	
Veratric acid	H	OCH_3_	OCH_3_	H	
Salicylic acid	OH	H	H	H	
**Cinnamic Acids**	**R_1_**	**R_2_**	**R_3_**	**R_4_**	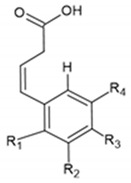
Cinnamic acid	H	H	H	H	
o-Coumaric acid	OH	H	H	H	
m-Coumaric acid	H	OH	H	H	
p-Coumaric acid	H	H	OH	H	
Ferulic acid	H	H	OH	OCH_3_	
Caffeic acid	H	OH	OH	H	

**Table 2:** LOD and LOQ values of CGA for all three electrodes [54].

Electrode	LOD (M)	LOQ (M)
C-SPE	6.50 × 10^−7^	2.16 × 10^−6^
GPH-SPE	0.73 × 10^−7^	2.45 × 10^−6^
GPH-GNP-SPE	0.62 × 10^−7^	1.94 × 10^−7^

**Table 3:** Main electrochemical sensors and biosensors used for CGA detection.

Sensitive Material	Detection Technique	Linear Range µmol·L^−1^	LOD µmol·L^−1^	Real Samples	Ref.
**Sensors**					
MIS/Au	DPV	5 × 10^−1^–12	1.48 × 10^−1^	Coffee Tea	[59]
MWCNTs/SPE	CV, DPV	4.8 × 10^−1^–44.59	3.4 × 10^−1^	Coffee beans	[52]
Fe_3_O_4_@MIL-100(Fe)/GCE	EIS, CV	1 × 10^−1^–460	5 × 10^−2^	Apple Coffee	[60]
UiO-66-NH_2_/TiO_2_	CV, DPV	1 × 10^−2^–13.0	7 × 10^−3^	Coffee Tea	[61]
IL/DMC/PE	CV, EIS, SWV	2 × 10^−2^–2.5	1 × 10^−2^	Herbal extracts of *Calendula* *ocinalis* and *Echinacea purpurea*	[62]
TAPB-DMTP-COFs/AuNPs	CV, DPV	1 × 10^−2^–40	9.5 × 10^−3^	Coffee Apple Honeysuckle	[63]
LGN-MWCNTs-CuONPs-GCE	CV, DPV	5–50	1.25 × 10^−2^	Coffee	[64]
NbNPs/CNTs/GCE	CV, SWV	2 × 10^−3^–2	8.2 × 10^−4^	Coffee Tomato Drinks	[67]
PLE	SWV	7.7 × 10^−2^–7.7	4.5 × 10^−3^	Clinical samples (Human urine)	[68]
GPH-GNP-SPE	CV	1 × 10^−1^–1.20	6.2 × 10^−2^	Nutraceutical products based on Green Coffee extract	[58]
**Biosensors**					
Bean sprout homogenate	SWV	3.48–320	8 × 10^−1^	Coffee	[75]
Ir-BMI.PF_6_-PPO	SWV	3.48–49.50	9.1 × 10^−1^	Decaffeinated coffee	[76]
C-SPE/Pt-NPs/RGO/Lacc	CV, CA	2.91–26.47	2.67	Coffee	[77]
GCE-CO-LAC-MWCNTs	CV, CA	1–300	3 × 10^−1^	Juice	[78]
Lap/{[(VBT)(VBA)_4_]^4+^} ≈ _25_/AuNP/HRP	CV, EIS	1–120	2.7 × 10^−3^	Yerba mate Green Coffee	[79]
Cu(II)-Nc protein-based biosensor	Colorimetric detection	25.0−250	1.2	Herbal plant extracts (sage, green tea, mint, and marjoram)	[80]

MIS/Au—molecularly imprinted siloxane/Au sensor; DPV—differential pulse voltammetry; MWCNTs/SPE—multi-walled carbon nanotubes modified screen-printed electrode; CV—cyclic voltammetry; Fe_3_O_4_@MIL-100(Fe)/GCE—superparamagnetic Fe_3_O_4_ core encapsulated into a metal–organic framework shell/glassy carbon electrode; EIS—electrochemical impedance spectroscopy; UiO-66-NH_2_/TiO_2_—metal–organic frameworks/titanium dioxide; IL/DMC/PE—highly defective mesoporous carbon–ionic liquid paste electrode; SWV—square wave voltammetry; TAPB-DMTP-COFs/AuNPs—1,3,5-tris(4-aminophenyl)benzene- 2,5-dimethoxyterephaldehyde- covalent organic frameworks/gold nanoparticles; LGN-MWCNTs-CuONPs-GCE–lignin polymer-multiwalled carbon nanotubes-copper oxide nanoparticles-glassy carbon electrode; NbNPs/CNTs/GCE—glassy carbon electrode modified with niobium nanoparticles and multi-walled carbon nanotubes; PLE—pencil lead electrode; GPH-GNP-SPE—screen-printed electrode modified with graphene and gold nanoparticles; Ir-BMI.PF_6_-PPO—1-n-butyl-3-methylimidazolium hexafluorophosphate containing dispersed iridium nanoparticles (Ir-BMI.PF6) and polyphenol oxidase; C-SPE/Pt-NPs/RGO/Lacc—carbon screen-printed electrode modified with platinum nanoparticles, reduced graphene oxide and laccase; CA—chronoamperometry; GCE-CO-LAC-MWCNTs—glassy carbon electrode modified with graphene oxide, laccase and multi-walled carbon nanotubes; Lap/{[(VBT)(VBA)_4_]^4+^} ≈ _25_/AuNP/HRP—horseradish peroxidase immobilization onto nanohydrogels made of laponite, gold nanoparticles and a vinylbenzyltriethylammonium polycation copolymerized with vinylbenzylthymine groups; Cu(II)-Nc protein-based biosensor—protein-based solid prooxidant neocuproine biosensor for determining the Cu(II)-induced prooxidant activity.
